# Extract of *Silybum marianum* (L.) Gaertn Leaves as a Novel Green Corrosion Inhibitor for Carbon Steel in Acidic Solution

**DOI:** 10.3390/ma17194794

**Published:** 2024-09-29

**Authors:** Yubin Wang, Lingjie Li, Jinbei He, Baojiang Sun

**Affiliations:** 1 School of Petroleum Engineering, China University of Petroleum (East China), Qingdao 266580, China; 2 CNPC Engineering Technology Research Co., Ltd., Tianjin 300451, China; 3 Hubei Jingyu Material Co., Ltd., Wuhan 430073, China

**Keywords:** corrosion, corrosion inhibitor, *Silybum marianum* (L.) Gaertn, adsorption

## Abstract

In this work, leaves of *Silybum marianum* (L.) Gaertn were extracted by a one-step extraction method using ethanol as a solvent, and the *Silybum marianum* (L.) Gaertn extract (SMGE) was firstly employed as a green corrosion inhibitor for carbon steel in 0.5 mol/L H_2_SO_4_. The corrosion inhibition performance was studied using weight loss and electrochemical methods, and the anti-corrosion mechanism of SMGE is further analyzed through some surface characterizations and theoretical calculations. The results indicate that SMGE can act as a mixed-type corrosion inhibitor and possess superior corrosion inhibition performance for carbon steel in H_2_SO_4_ solution, and the optimum corrosion inhibition efficiency reached 93.06% at 800 ppm SMGE combined with 60 ppm KI. The corrosion inhibition efficiency increased with the rising inhibitor concentration. Surface characterizations illustrated that the inhibitor could physico-chemically adsorb on a metal surface, forming a hydrophobic, protective film.

## 1. Introduction

The corrosion phenomenon is a spontaneous behavior of metal materials [1] which results in huge economical losses and many safety issues [2]. Corrosion control techniques can save more than one-third of corrosion costs [3]. The corrosion inhibitor method, as one of most commonly anti-corrosion techniques in the closed system, is widely applied in industrial fields [4,5,6,7], like cooling water systems in nuclear power plants, oil production and transportation pipelines, drinking water pipelines, coatings, pickling, battery systems, etc. This method utilizes efficient corrosion inhibitors to protect metal from corrosion and possesses some remarkable advantages, including easy operation, low cost, high efficiency, etc. Traditional corrosion inhibitors can be classified as inorganic and organic types; among them, inorganic corrosion inhibitors mainly consist of some inorganic salts, like chromate, nitrite, phosphate, molybdate, tungstate, etc. The inherent toxicity of most inorganic corrosion inhibitors to human health and the environment makes it difficult to satisfy the environmental requirements [5]. Additionally, to achieve enough corrosion inhibition capability, the dosage of inorganic corrosion inhibitors is very large, thereby increasing the usage cost. Organic-type corrosion inhibitors, like imidazolines, Mannich bases, and quaternary ammonium salts, are generally composed of N, S, O, or other heteroatoms for adsorbing on metal surfaces. Generally, the corrosion inhibition performance of organic corrosion inhibitors is better than inorganic corrosion inhibitors with the same dosage [4]. However, the residue of organic corrosion inhibitors in waste water can cause eutrophication, which is harmful for the environment. With the increasing awareness of sustainable development, developing eco-friendly corrosion inhibitors is of great importance, and has become one of the current research hotspots.

In recent decades, a number of eco-friendly corrosion inhibitors have been reported, including plant-extracted corrosion inhibitors [6], expired drugs, ionic liquids, functional nanomaterials, rare earth, etc. Like the above-mentioned types, corrosion inhibitors from plant-extracted products have attracted widespread attention. S. Wan et al. developed several plant extracts as corrosion inhibitors for carbon steel in acidic media, including soybean extract [7], kapok leaf extract [8], Acanthopanax senticosus leaf extract [9], Fructus cannabis protein extract [10], and parsley extract [11]. These reported corrosion inhibitors have displayed quite good corrosion inhibition efficiency. B. Liao et al. [12] studied protein waste extract as a corrosion inhibitor for carbon steel in acidic solution, and the highest efficiency reached 96.2%. B. Tan et al. [13] investigated pyracantha fortuneana alcohol extracts as a mix-type corrosion inhibitor for copper in H_2_SO_4_ solution, and its corrosion inhibition efficiency was more than 95% with the addition of 600 ppm. The widely distributed cheap resources and biodegradable properties endow plant-extracted corrosion with advantages.

*Silybum marianum* (L.) Gaertn, as a traditional medicinal plant, can be utilized to treat some diseases, with anti-cancer and anti-diabetic effects [14]. As reported [15,16,17], compositions of *Silybum marianum* (L.) Gaertn consist of O, N-containing groups, including a large content of silymarin, a mixture of three flavonolignans (silybin, silydianin and silycristin) [18], fatty acids (mainly including linoleic, linolenic, myristic, oleic, palmitic, and stearic acid), protein, tocopherol, and sterols (mainly including cholesterol, campesterol, stigmasterol, and sitosterol). It is native to the Mediterranean regions and is now widely distributed in China, Europe, North Africa, Iran, etc. [19,20]. It is called various names, like milk thistle, Mary thistle, etc. Based on previous reports, the flavonolignans-containing plant extracts generally exhibit superior corrosion inhibition performance [21,22]; thereby, extracts of *Silybum marianum* (L.) Gaertn possess promising potential corrosion inhibition ability. However, to date, limited work on *Silybum marianum* (L.) Gaertn as a corrosion inhibitor can be found.

In this work, a one-step extraction method of organic compounds from *Silybum marianum* (L.) Gaertn leaves is proposed. The obtained extract (SMGE) was evaluated as a corrosion inhibitor through gravimetric and electrochemical measurements. The corroded surfaces of the specimens were analyzed by combining several techniques, such as optical microscopy (OM), water contact angle (WCA), and X-ray photoelectron spectroscopy (XPS). Based on the obtained results, the corrosion inhibition mechanism of SMGE is proposed. The significance of this study lies not only in providing a novel corrosion inhibitor candidate for the field of metal corrosion protection in acidic media, but also in promoting further research and applications of *Silybum marianum* (L.) Gaertn waste leaves as naturally derived sources for developing eco-friendly corrosion inhibitors.

## 2. Experimental

### 2.1. Preparation of Materials

**Figure 1:**
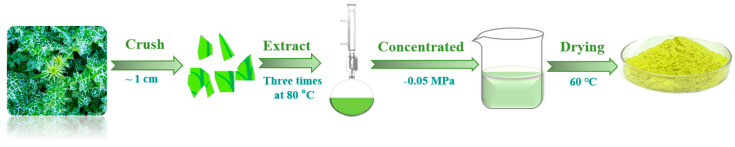
Schematic diagram for preparation of SMGE.

*Silybum marianum* (L.) Gaertn leaves were purchased from Yishan Traditional Chinese Medicine Co., Ltd. in the Guangxi province of China. SMGE was prepared using the pure ethanol extraction method. As shown in Figure 1, leaves were firstly washed using deionized water, dried at 40 °C in an oven, and then crushed into pieces with a size of 0.5~1 cm. The crushed leaves were extracted using ethanol through the Soxhlet extraction method three times at 80 °C. The solution was then concentrated under −0.05 MPa and dried at 60 °C to obtain SMGE powders.

Carbon steel species with 10 mm length × 10 mm width × 5 mm height consisted of 0.22 wt.% C, 1.4 wt.% Mn, 0.045 wt.% S, 0.35 wt.% Si, and Fe as the rest. As for the preparation of working electrode, a copper wire was soldered on the back of steel to ensure electric connection and then encapsulated using epoxy resin, leaving an exposed area of 1 cm^2^. The exposed surface was sanded using 150, 400, 800, and 1200 grit SiC papers, then cleaned using acetone and dried through cold air. All solutions were prepared using analytical reagents and deionized water.

### 2.2. Mass Loss Measurement

The mass loss method was utilized to evaluate the corrosion rate of metal. Steel coupons were prepared and weighted using an analytical balance before and after immersion for 6 h. The corroded samples were cleaned and dried according to the ASTM G103 [23] recommendation. Corrosion inhibition efficiency (η*_w_*) was further calculated from weight loss values using Equations (1) and (2).

(1) $$V=87.6\times \frac{\Delta W}{S\times \rho_{m}\times t}$$

(2) $$\eta_{w}\left(\%\right)=\frac{V_{0}-V}{V_{0}}\times 100$$

where *V* represents the metal corrosion rate; Δ*W* is the weight loss (mg); S is the exposed area of the sample; *ρ_m_* is the sample density (7.28 g/cm^3^); and V_0_ and V represent the corrosion rate in the absence (blank solution) and presence of inhibitors, respectively.

### 2.3. Electrochemical Measurements

A traditional three-electrode cell was utilized to conduct electrochemical tests using an electrochemical Corrtest electrochemical workstation, including open circuit potential (OCP), electrochemical impedance spectroscopy (EIS), and potentiodynamic polarization (PDP). The encapsulated steel sample acted as a working electrode with a working area of 1 cm^2^; a platinum plate with an exposed area of 2 cm^2^ acted as a counter electrode; and the saturated calomel electrode (SCE) was used as a reference electrode. Before each electrochemical test, the OCP test was carried out for 1800 s to achieve a stable potential state. Then, the EIS test was conducted with an applied amplitude of 10 mV for the sinusoidal signal within a frequency range from 10^−2^ to 10^4^ Hz. The PDP measurement was performed from −0.25 to +0.25 V versus OCP at a scanning rate of 0.5 mV/s. ZView 3.3a and CView software 3.5c were applied to fit the relevant electrochemical parameters from EIS and PDP results, respectively.

### 2.4. Surface Characterizations

The morphologies of inhibited and corroded metal surfaces were observed using optical microscopy (OM), and the corresponding hydrophobic surface parameters were measured using the Water Contact Angle Tester (WCA, ZJ-7000, Z. Jia Equipment, Shenzhen, China). X-ray Photoelectron Spectroscopy (XPS, AXIS NOVA, KRATOS Co., Ltd. Japan) was carried out to analyze the compositions of corrosion products, with the binding energy scale standardized to C 1s peak at 284.8 eV as a reference value for the calibration of other elemental peak positions.

### 2.5. Theoretical Calculations

In order to obtain the quantum chemical parameters of corrosion inhibitor molecules, the theoretical calculation based on the method of density functional theory (DFT) was carried out using the commercial software Materials Studios (MS 2023) [8]. As previously reported [4,16], geometric optimization and single-point energy calculation were carried out at a Becke, 3-parameter, Lee–Yang–Parr (B3LYP) functional basis level, including the frontier molecular orbital energy and electrostatic potential. Among these, the frontier molecular orbital energy contained the highest occupied molecular orbital energy (E_HOMO_) and the lowest unoccupied molecular orbital energy (E_LUMO_).

## 3. Results and Discussion

### 3.1. Corrosion Inhibition Performance

Table 1 lists the change in metal mass prior to and after the weight loss test, according to Equations (1) and (2), the corresponding corrosion inhibition efficiency η*_w_* was obtained. In the blank 0.5 mol/L H_2_SO_4_ solution, the value of metal mass loss reached 0.2137 g after 6 h of immersion, indicating that the metal suffered from severe corrosion under this condition. With the addition of 60 ppm KI, the value of weight loss slightly decreased, which was equal to 0.1831 g, illustrating that KI could not effectively retard metal corrosion. In the inhibitor-containing solutions, the value of weight loss dramatically decreased compared with that in blank solution. Additionally, the weight loss amount decreased with the rising MSGE concentration, and the corresponding η*_w_* increased. As the concentration of MSGE was 800 ppm and that of KI was 60 ppm, the value of weight loss decreased to 0.033 g and the value of η*_w_* reached 84.52%, showing the quite good corrosion inhibition ability for carbon steel under acidic conditions.

**Figure 2:**
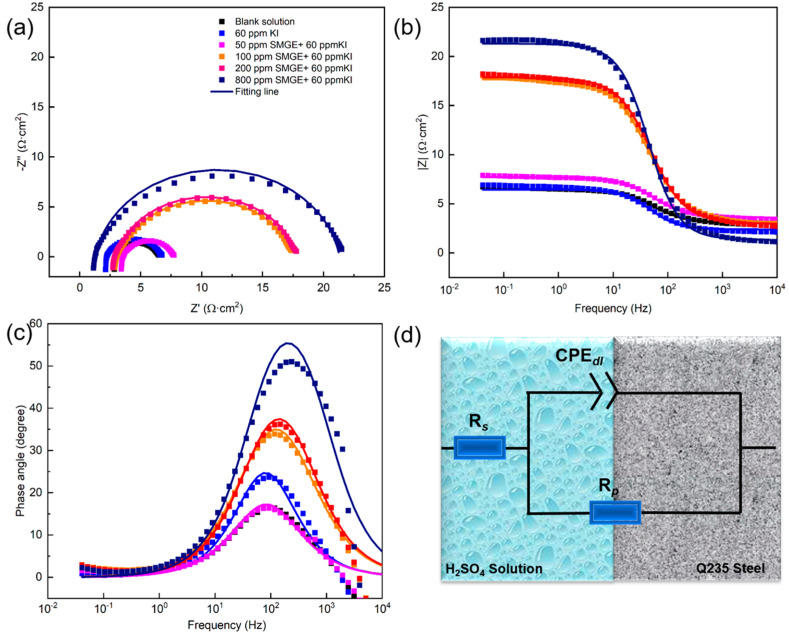
Nyquist (**a**) and Bode (**b**,**c**) plots of EIS spectra, and the equivalent circuit diagram (**d**) for carbon steel in 0.5 mol/L H_2_SO_4_ solutions containing different concentrations of SMGE.

**Figure 3:**
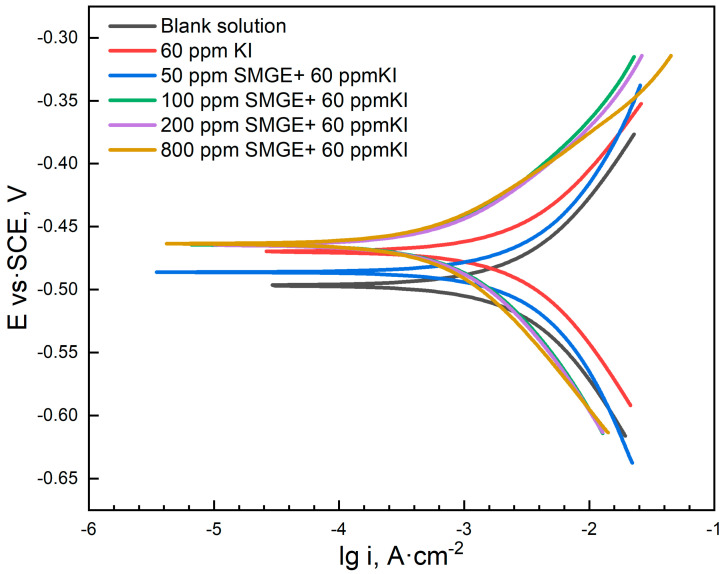
PDP plots for carbon steel in the absence and presence of different inhibitor concentrations.

EIS and PDP measurements were performed to further study the corrosion inhibition behavior of SMGE combined with KI, as shown in Figure 2 and Figure 3. The equivalent circuit diagram in Figure 2d was utilized to fit the relevant electrochemical parameters; among them, R*_s_* and R*_p_* refer to the resistance of the solution between the reference electrode and working electrode and the polarization resistance, respectively. Generally, the value of R*_p_* was reverse proportional to the corrosion rate. CPE was the constant phase element, representing the capacitance characteristic due to the non-ideal behaviour in the interface between the metal and the solution [24]. CPE*_dl_*-T indicates the interface double electric layer capacitance and n indicates the phase shift, which was determined by the inhomogeneity of the interface. σ^2^ was the chi-squared between the experimental and fitting data, and its value was lower than 0.01, indicating the quite accurate fitting level [25]. The obtained fitted parameters are displayed in Table 2. The value of R*_p_* became large with the addition of inhibitors, and it increased with the rising inhibitor concentration. In the absence of inhibitors, the value of R*_p_* was 3.50 Ω·cm^2^; with the addition of 800 ppm SMGE + 60 ppm KI, it increased to 22.96 Ω·cm^2^. The increase in R*_p_* proved that the inhibitor molecules could effectively retard metal corrosion behaviour. Moreover, the corrosion inhibition efficiency (η*_EIS_*) based on the EIS test could be calculated based on Equation (3).

(3) $$\eta_{EIS}\left(\%\right)=\frac{R_{p}-R_{p}^{0}}{R_{p}}\times 100$$

where *R_p_* and *R*^0^*_p_* represent the polarization resistance with and without the inhibitor, respectively. η*_EIS_* increased with the increase in the inhibitor dosage, and it reached 84.74% with the addition of 800 ppm SMGE combined with 60 ppm KI, which agreed well with the results from the weight loss method. Additionally, the value of CPE gradually decreased with the addition of the inhibitor, which could have been caused by the adsorption of inhibitor molecules on the metal/solution interface, which replaced water molecules, increasing the thickness of the electric double layer and decreasing the local dielectric constants.

PDP curves are shown in Figure 3 and were fitted using the Tafel extrapolation method. The obtained parameters are shown, including the corrosion potential (E*_corr_*), corrosion current density (i*_corr_*), as well as the apparent anodic (β*_a_*) and cathodic (β*_c_*) Tafel slopes. And the corrosion inhibition efficiency from PDP test (η*_p_*) was calculated using the following Equation (4):

(4) $$\eta_{p}\left(\%\right)=\frac{i_{corr}^{0}-i_{corr}}{i_{corr}^{0}}\times 100$$

where $i_{corr}^{0}$ and $i_{corr}$ represent the corrosion current density in the absence and presence of the ODH inhibitor, respectively. As shown in Table 3, compared with the value of corrosion current density i_0_ in blank solution, it decreased significantly with the addition of the inhibitor. And the larger the inhibitor dosage, the smaller the corrosion current density. The corresponding η*_p_* increased with the rising inhibitor dosage, and it reached 93.06% with the addition of 800 ppm SMGE and 60 ppm KI, exhibiting quite high corrosion inhibition ability. There existed no obvious change in the value of E*_corr_*, indicating that the inhibitors belonged to the mixed-type corrosion inhibitor, affecting both cathodic and anodic behaviors. Weight loss and electrochemical measurements illustrated that SMGE combined with KI could effectively inhibit the corrosion behavior of carbon steel in H_2_SO_4_ solution, and the corrosion inhibition performance became better with the rising inhibitor concentration.

### 3.2. Morphology and Composition of Corrosion Products

**Figure 4:**
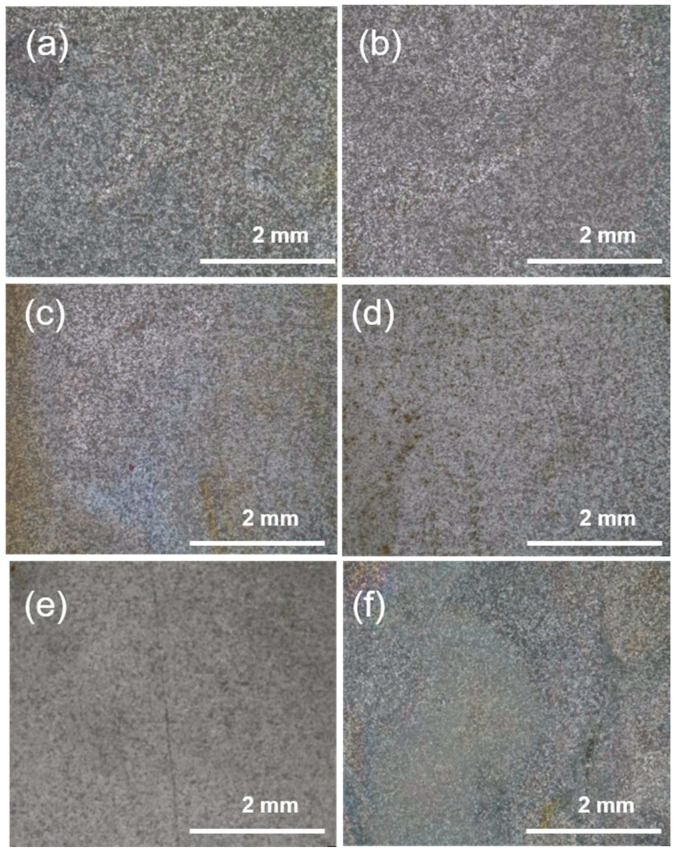
OM observation of corroded carbon steel after immersion in 0.5 mol/L H_2_SO_4_ solution for 6 h without or with inhibitors: 0 (**a**), 60 ppm KI (**b**), 50 ppm SMGE + 60 ppm KI (**c**), 100 ppm SMGE + 60 ppm KI (**d**), 200 ppm SMGE + 60 ppm KI (**e**), 800 ppm SMGE + 60 ppm KI (**f**).

**Figure 5:**
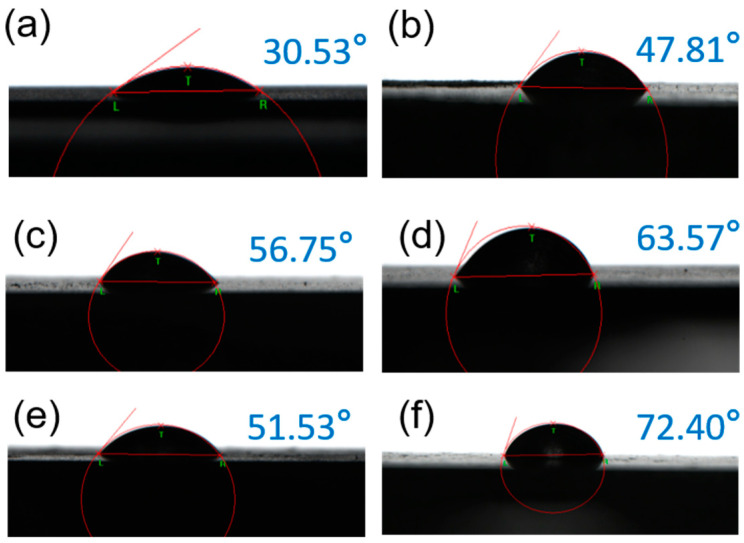
WCA measurements of corroded surface for carbon steel after immersion in 0.5 mol/L H_2_SO_4_ solution for 6 h without or with inhibitors: 0 ppm (**a**), 60 ppm KI (**b**), 50 ppm SMGE+ 60 ppm KI (**c**), 100 ppm SMGE + 60 ppm KI (**d**), 200 ppm SMGE + 60 ppm KI (**e**), 800 ppm SMGE + 60 ppm KI (**f**).

Figure 4 and Figure 5 show OM images and the corresponding WCA results. As shown in Figure 4, the metal surface was uniformly covered with corrosion products in yellow or grey color, which could be iron oxides and/or hydroxides (Figure 4a). The amount of corrosion products decreased with the addition of inhibitors, and a film could be observed at a larger dosage (Figure 4f). The value of WCA increased with the rising inhibitor concentration. It increased from 30.53° to 72.40° at 800 ppm of inhibitor, indicating that the protective film possessed hydrophobicity, which could retard the invasion of water-soluble corrosive species, like H^+^, dissolved O_2_, etc. Thus, this hydrophobic film was beneficial for corrosion protection.

**Figure 6:**
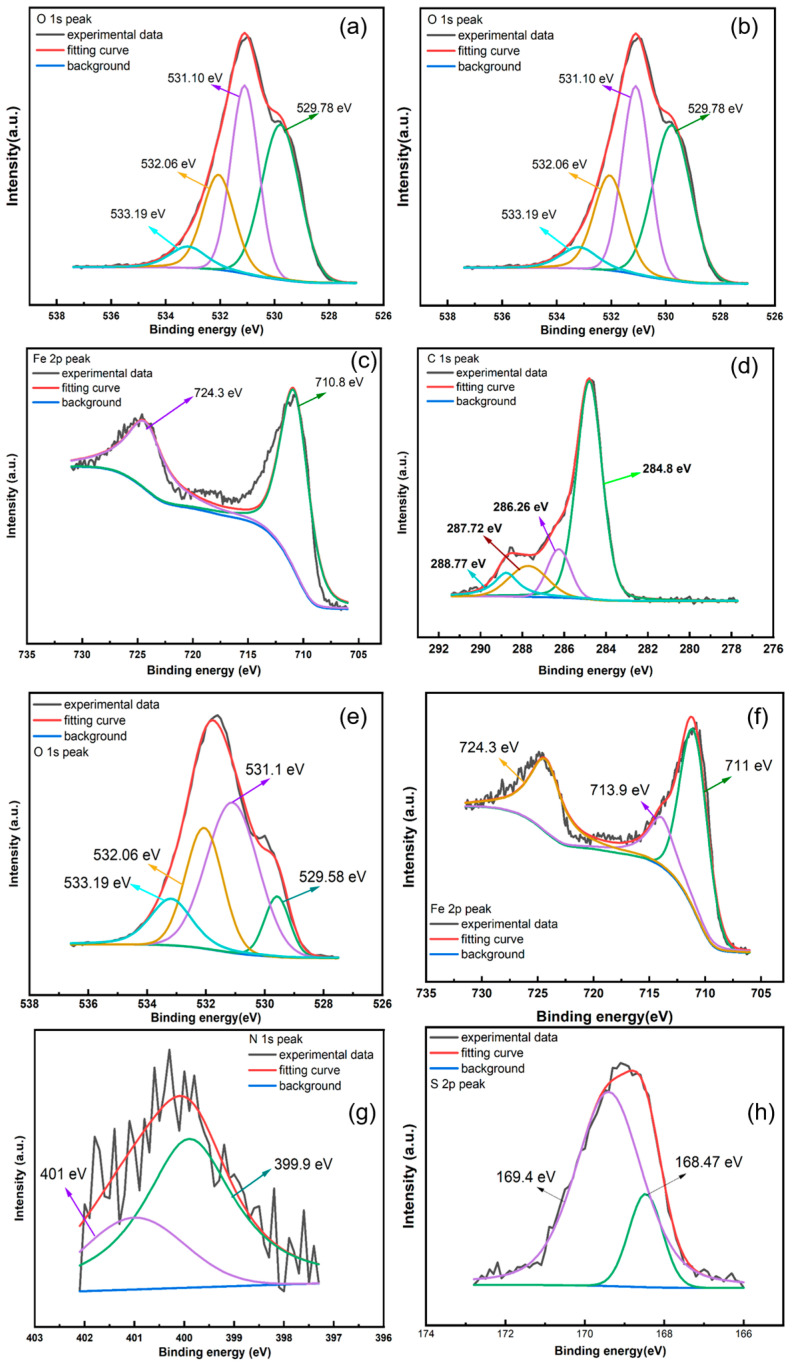
XPS spectra of corrosion products on carbon steel surface after 6 h immersion in 0.5 mol/L H_2_SO_4_ solution without (**a**–**c**) and containing (**d**–**h**) 800 ppm SMGE + 60 ppm KI.

XPS tests were conducted to analyze the compositions of corrosion products in the absence and presence of inhibitors, as shown in Figure 6. In the absence of inhibitors, the corrosion products mainly consisted of C, O, and Fe elements. The C 1s spectrum (Figure 6a) exhibited three peaks [26] corresponding to C-H at 284.80 eV, C=O at 287.70 eV, and C-OH/C-O-C bonds at 286.26 eV [27]. The Fe 2p spectrum (Figure 6b) could be decomposed into two peaks at 710.8 and 724.3 eV, which could be attributed to Fe Fe2p_3/2_ and Fe2p_1/2_, and the Fe element mainly existed in the +3 state [28,29]. The O 1s spectrum (Figure 6c) contained peaks corresponding to oxygen in metal oxides at 529.78 and 531.10 eV, O-C at 533.19 eV, and hydroxyl or water molecules at 532.06 eV [30]. Upon the addition of 800 ppm SMGE + 60 ppm KI, N 1s and S 2p high-resolution spectra appeared, while an additional peak emerged in the C 1s spectrum, indicating that N- and S-containing corrosion products formed and inhibitor molecules adsorbed onto the metal surface and/or corrosion products. The C 1s spectrum (Figure 6d) revealed four peaks corresponding to C-O or C=O at 286.26 eV, C-C at 284.80 eV, C-N at 287.72 eV, and C-N^+^ at 288.77 eV. The Fe 2p spectrum (Figure 6f) exhibited three peaks: Goethite at 711 eV, Fe(III) formal oxidation state at 713.9 eV [31,32,33], and the peak of 724.3 eV, which implied the existence of Fe-N-C compounds [34]. Peaks in the O 1s spectrum (Figure 6e) were ascribed to Fe-O-Fe at 529.58 eV, hydroxyl or water molecules at 532.06 eV, O-C at 533.19 eV, and O-Fe bond at 531.10 eV. The N 1s spectrum (Figure 6g) exhibited two peaks: the peak at 399.90 eV was assigned to N-Fe [35] and the −N-H- at 401.00 eV [36]. The S 2p spectrum contained two peaks, including oxygen-containing S group at 169.40 eV [37] and sulfur in the sulfate at 168.47 eV [38,39,40,41]. XPS results confirmed that the inhibitor molecules could adsorb onto the metal surface through chemical adsorption.

### 3.3. Corrosion Inhibition Mechanism

**Figure 7:**
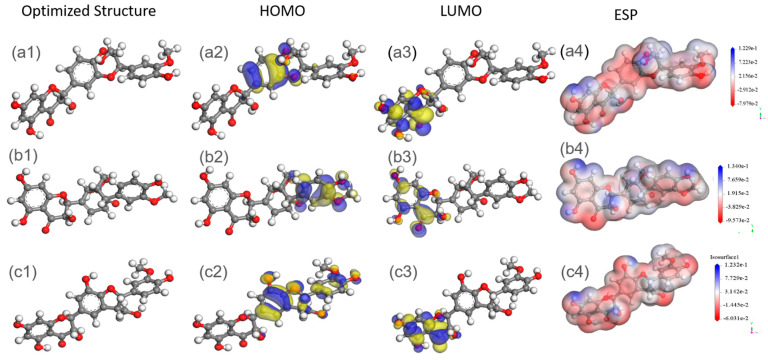
Optimized geometry structures; HOMO, LUMO, and ESP diagrams for Silybin (**a1**–**a4**), Silydianin (**b1**–**b4**), and Silychristin (**c1**–**c4**).

In H_2_SO_4_ solution, electrochemical corrosion reactions for carbon steel mainly included dissolution reactions of iron as anodic reactions and hydrogen evolution reactions as cathodic reactions. Severe corrosion behavior of carbon steel resulted in significant weight loss, as shown in Table 1. With the addition of inhibitors, namely, SMGE combined with KI, inhibitor molecules could adsorb onto the metal surface through physical and chemical adsorption (see from XPS results), forming a protective film (Figure 4) and thus retarding the invasion of corrosive species. Additionally, as shown in Figure 7, we calculated the optimized geometry structures; the highest occupied molecular orbital energy (*E*_HOMO_); the lowest unoccupied molecular orbital energy (*E*_LUMO_); and the electrostatic potential distribution (ESP) of Silybin, Silydianin, and Silychristin using MS 2023 software. The energy gap (∆E) between E_LUMO_ and E_HOMO_ is also an important factor for judging the stability of organic molecules. A low ∆E value indicates that the corrosion inhibitor molecule is more reactive and easier to adsorb onto the metal surface, as well as more efficient at corrosion inhibition [11]. Compared with the value of the energy gap in some recent work [4,10,12], the values of ∆E for the main compositions of SMGE were 2.6657, 1.3537, and 2.7151 eV, respectively. The lower values illustrated the higher corrosion protection ability of SMGE [8]. As for ESP diagrams, the red and blue regions indicate the nucleophilic and electrophilic activities of molecules [9], respectively. The red regions for the calculated molecules were mainly distributed on oxygen atoms, which were beneficial for the formation of chemical bonds with Fe atoms.

As shown in the EIS spectra (Figure 2), the increase in R*_p_* strongly proved that inhibitor molecules could effectively retard the corrosion process. Additionally, according to the PDP curves in Figure 3, the decrease in the corrosion current density further confirmed the superior corrosion inhibition performance of the inhibitors, which belonged to the mixed-type corrosion inhibitor. WCA characterization illustrated that the protective adsorption film was hydrophobic and could enhance the corrosion inhibition ability of inhibitors.

## 4. Conclusions

In this work, the *Silybum marianum* (L.) Gaertn extract (SMGE) was first prepared and utilized as a novel green corrosion inhibitor for carbon steel in 0.5 mol/L H_2_SO_4_ solution from waste leaves of *Silybum marianum* (L.) Gaertn. The corrosion inhibition performance and relevant mechanism of SMGE were studied through electrochemical tests, surface characterizations, and theoretical calculations. The following conclusions can be drawn:

- Waste leaves of *Silybum marianum* (L.) Gaertn can be utilized as raw materials to prepare novel green corrosion inhibitors for carbon steel in 0.5 mol/L H_2_SO_4_.
- SMGE showed superior corrosion inhibition ability for carbon steel in H_2_SO_4_ solution. The corrosion inhibition efficiency increased with the inhibitor dosage, and the maximum value reached 93.06% with the addition of 800 ppm SMGE and 60 ppm KI.
- SMGE can act as a mixed-type corrosion inhibitor and physico-chemically adsorb onto a metal surface, forming a hydrophobic protective film, thus protecting carbon steel from corrosion. The adsorption sites of inhibitor molecules for chemical bonding were mainly distributed on heteroatoms.

## Figures and Tables

**Table 1:** Corrosion inhibition efficiency obtained from weight loss method for different MSGE concentrations mixed with 60 ppm KI.

Concentration of Corrosion Inhibitor	W1 (g)	W2 (g)	∆W (g)	η*_w_* (%)
0	11.3388	11.1251	0.2137	--
60 ppm KI	11.2362	11.0531	0.1831	14.05
50 ppm SMGE + 60 ppm KI	11.2772	11.2063	0.0709	65.64
100 ppm SMGE + 60 ppm KI	11.3704	11.3218	0.0486	76.14
200 ppm SMGE + 60 ppm KI	11.3749	11.3312	0.0437	78.92
800 ppm SMGE + 60 ppm KI	11.3283	11.2952	0.0330	84.52

**Table 2:** Fitted parameters from EIS curves in Figure 2.

Concentration of Corrosion Inhibitor	R*_s_* (Ω·cm^2^)	CPE*_dl_* − T (S^n^·Ω^−1^·cm^−2^)	n	*R_p_* (Ω·cm^2^)	σ^2^	η*_EIS_* (%)
0	2.51	0.0020231	0.85	3.50	6.63 × 10^−4^	-
60 ppm KI	2.38	0.0012002	0.93	4.04	4.63 × 10^−4^	13.36
50 ppm SMGE + 60 ppm KI	3.19	0.0016129	0.86	4.11	1.08 × 10^−4^	14.91
100 ppm SMGE + 60 ppm KI	3.12	0.00061973	0.84	14.55	4.84 × 10^−4^	75.92
200 ppm SMGE + 60 ppm KI	2.94	0.00048374	0.86	14.94	1.93 × 10^−4^	76.55
800 ppm SMGE + 60 ppm KI	2.11	0.00018701	0.93	22.96	2.47 × 10^−4^	84.74

**Table 3:** Fitted parameters for PDP results.

Concentration of Corrosion Inhibitor	β*_a_* (mV/dec)	β*_c_* (mV/dec)	*i_corr_* (A/cm^2^)	*E_corr_* (V vs. SCE)	η*_PDP_* (%)
0	244	−282	8.4718 × 10^−3^	−0.4967	--
60 ppm KI	147	−171	4.2634 × 10^−3^	−0.4699	49.68
50 ppm SMGE + 60 ppm KI	173	−189	3.8892 × 10^−3^	−0.4878	54.09
100 ppm SMGE + 60 ppm KI	105	−140	1.1764 × 10^−3^	−0.4622	86.11
200 ppm SMGE + 60 ppm KI	102	−135	1.1443 × 10^−3^	−0.4665	86.49
800 ppm SMGE + 60 ppm KI	86	−121	5.8882 × 10^−4^	−0.4911	93.06

## Data Availability

The original contributions presented in the study are included in the article, further inquiries can be directed to the corresponding authors.
